# Hip Reconstruction in Children with Cerebral Palsy: Comparing Treatment Plans Derived from Pelvic Radiographs Versus Those from Hip CTs

**DOI:** 10.3390/jcm15062259

**Published:** 2026-03-16

**Authors:** Andy Tsai, Patrick Johnston, Benjamin J. Shore

**Affiliations:** 1 Department of Radiology, Boston Children’s Hospital, Harvard Medical School, Boston, MA 02130, USA; andy.tsai@childrens.harvard.edu (A.T.); patrick.johnston@childrens.harvard.edu (P.J.); 2 Department of Orthopedics, Boston Children’s Hospital, Harvard Medical School, Boston, MA 02130, USA

**Keywords:** cerebral palsy, computed tomography, hip displacement, hip surgery, migration percentage

## Abstract

**Background/Objectives**: Hip displacement is a common problem in children with cerebral palsy (CP). Typically, the recommended hip surveillance imaging for these children consists of an anteroposterior pelvic radiograph, from which we calculate the migration percentage (MP) to determine treatment plans (conservative/preventive therapy, femoral osteotomy, femoral and pelvic osteotomies, and salvage surgery). However, little is known about the accuracy of MP for treatment planning. We aim to compare treatment plans based on MP thresholds with plans determined by an orthopedic surgeon following review of the hip CTs. **Methods**: We retrospectively identified hip CTs performed in children who were ≤18 years old with CP (11/2018—07/2024). The inclusion criteria were: (1) a pelvic radiograph performed 6 months prior to the hip CT; and (2) no surgeries between the pelvic radiograph and the hip CT. These hip CTs were randomized and blindly reviewed by an orthopedic surgeon to determine each child’s treatment plan (CT-treatment). Separately, a pediatric radiologist blindly reviewed the randomized pelvic radiographs and measured each hip’s MP to determine each child’s treatment plan (XR-treatment). We used kappa-agreement and Bland–Altman analyses to compare XR- and CT-treatments. **Results**: Our study cohort consisted of 139 children (mean age = 9.3 ± 3.8 years; male = 90) with 278 hips. The proportion of agreement and unweighted kappa between XR- and CT-treatment were both low: 0.532 (148/278) and 0.339, respectively. Bland–Altman analyses showed that XR-treatment and CT-treatment were exchangeable when MP ≤ 10% but were not exchangeable otherwise. **Conclusions**: We should be cautious about relying exclusively on pelvic radiographs and subsequent MP calculation in making treatment decisions for hip displacement in children with CP since many anatomic details become evident on 3D imaging.

## 1. Introduction

Cerebral palsy (CP) is a group of disorders that affect movement, balance, and posture caused by damage to the developing brain before or after birth [1,2,3,4,5]. Of the many musculoskeletal problems that affect children with CP, hip displacement is the second most common [6,7,8,9,10,11]. As such, surveillance hip imaging, consisting of a single anteroposterior (AP) pelvic radiograph, is recommended every 6–12 months depending on the child’s ambulatory status or gross motor function classification system (GMFCS) level [12,13,14,15,16,17]. Scalar metrics derived from these pelvic radiographs are commonly used to assess the structural integrity of the hips [18]. The most widely used metric is Reimer’s migration percentage (MP) [19], which measures the amount of the femoral head that falls outside the containment of the acetabulum in the coronal plane. This metric has been fundamental in guiding children’s treatment plans [20]. Based on experience and the literature [21,22,23,24,25,26,27,28], we and many other institutions have used the following MP thresholds to guide treatment: conservative/preventive therapy for 0% ≤ MP ≤ 40%; femoral osteotomy for 40% < MP ≤ 60%; femoral and pelvic osteotomies for 60% < MP ≤ 80%; and salvage surgery for 80% < MP ≤ 100%.

Traditionally, hip surgeons rely on pelvic radiographs and the derived MPs for treatment planning. However, the reliability of radiographic hip evaluation is limited [29,30]. As CP patients are medically complex and formulating surgical treatment plans for their hips is challenging, a growing number of hip surgeons are utilizing CTs to provide a more comprehensive and accurate assessment of the hip’s 3D bony pathomorphology. Therefore, the objective of this study was to compare the surgical guidance derived from two imaging approaches: the traditional, simplified metric-based approach using MP derived from pelvic radiographs versus a qualitative assessment approach based on 3D hip CTs. We aimed to evaluate the agreement between these imaging-based recommendations, recognizing that the final surgical planning is multifactorial and integrates these imaging findings with clinical examination and patient-specific factors.

## 2. Materials and Methods

This retrospective study was approved by our institutional review board and was compliant with HIPAA guidelines. The requirement for written informed consent was waived.

### 2.1. Study Cohort

We retrospectively reviewed our image archive to identify hip CTs performed in children who were ≤18 years old with CP (11/2018—07/2024). The inclusion criteria were: (1) screening AP pelvic radiograph performed within 6 months prior to the hip CT to ensure concurrent imaging; and (2) no surgeries between the pelvic radiograph and the hip CT. If a child had more than one paired imaging study during the study period, we included only the earliest pair.

### 2.2. Pelvic Radiographs

The AP pelvic radiographs were acquired using digital radiographic systems (Philips Healthcare, Cambridge, MA, USA) in accordance with the American College of Radiology guidelines [31]. The typical tube voltage and current-time were 70–80 kVp and 2.5–15 mAs, respectively. To minimize artifacts due to positioning, we had the child lie supine on the tabletop with the patellae facing upwards, and we placed paddings around the child to minimize the pelvic tilt and scoliosis [32].

### 2.3. Hip CTs

A Somatom Force scanner (Siemens, Erlangen, Germany) was used to obtain each child’s hip CT. We utilized dual-source scanning, a helical pitch of 3, weight-adjusted tube voltage, and automated tube current modulation. The field of view of the hip CTs was from the iliac crests to the knees (so that we could measure femoral version concurrently), though the femoral version measurements and the images of the mid-femurs to the knees were not part of this study.

### 2.4. Image Interpretation

**Figure 1:**
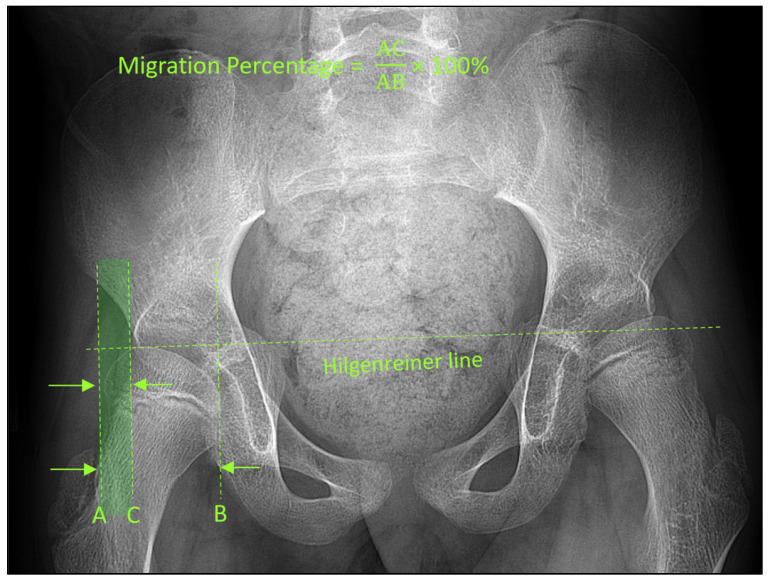
Pelvic radiograph from an 11-year-old boy with cerebral palsy. Several steps are required to compute the migration percentage (MP): (1) establish a Hilgenreiner line by drawing a line connecting the triradiate cartilages; (2) draw three perpendicular lines to the Hilgenreiner line, one abutting the lateral margin of the femoral head (A), one abutting the medial margin of the femoral head (B), and one abutting the lateral edge of the acetabular roof (C); (3) calculate the diameter of the femoral head by measuring the distance AB; and (4) calculate the amount of femoral head uncovered by measuring the distance AC. MP is the ratio of AC (lightly shaded region) to AB after converting it to percentages. In this child, the MP of the right hips was 25%.

A pediatric radiologist, with 15 years of post-fellowship experience in musculoskeletal imaging, blindly measured MPs from each patient’s AP pelvic radiograph (without access to their hip CTs). Each MP was measured in the standard fashion by first establishing a Hilgenreiner line followed by drawing three lines perpendicular to it: one abutting the medial femoral head, one abutting the lateral femoral head, and one abutting the lateral acetabulum (Figure 1). MP is the distance between the two lines abutting the lateral acetabulum and the lateral femoral head divided by the distance between the two lines abutting the medial and lateral femoral head multiplied by 100 (to change this ratio to a percentage). We then converted each of these MP measurements to one of four established treatment plans, based on the guiding MP thresholds defined above: [0, 40] for conservative/preventive therapy (score = 0), (40, 60] for femoral osteotomy (score = 1), (60, 80] for femoral and pelvic osteotomies (score = 2), and (80, 100] for salvage surgery (score = 3). We refer to this treatment plan as XR-treatment with scores of 0, 1, 2, or 3 for the purpose of subsequent statistical analysis. The pediatric radiologist’s role here was strictly to provide the standardized MP measurement from pelvic radiograph. The conversion of this measurement to a treatment category (XR-treatment) was an automatic step based on previously established treatment thresholds, isolating the metric from clinical interpretation.

Separately, a pediatric orthopedic surgeon with 15 years of post-fellowship experience in CP hip surgery blindly reviewed the patients’ hip CTs (without access to their pelvic radiographs). The CTs were analyzed using a picture archiving and communication system (Synapse, Fujifilm, Tokyo, Japan), which provided standard multiplanar reconstructions (axial, coronal, and sagittal planes) in addition to 3D volume-rendered reconstructions of the pelvis and proximal femora. The surgeon performed a qualitative, integrative assessment of the 3D bony morphology to determine the optimal treatment plan. Key anatomical features considered included acetabular depth, version, and sectoral coverage; femoral head sphericity and containment; the pattern of acetabular deficiency (anterior, posterior, or global); and femoral neck-shaft anatomy. Based on this synthesis, the surgeon assigned one of four treatment categories: conservative/preventive therapy (score = 0), femoral osteotomy (score = 1), femoral and pelvic osteotomies (score = 2), and salvage surgery (score = 3). We refer to this treatment plan as CT-treatment. This process represents the imaging input a surgeon would use during preoperative planning, simulating the 3D radiographic component of decision-making.

### 2.5. Statistical Analysis

We compared treatment plans y1 = XR-treatment and y2 = CT-treatment, each with scores of 0, 1, 2, or 3. A preliminary analysis was based on the 4 × 4 confusion matrix for y1 and y2 along with common kappa-based agreement indices: proportion of agreement, unweighted kappa, and (quadratically) weighted kappa.

Kappa-based agreement indices do not answer the question of exchangeability, namely, for a given hip, can the two methods be used interchangeably to determine treatment? To address this question, we used Bland–Altman methods, which are based on the concept of “tolerance intervals” (TIs) for the difference in scores, d = (y1 − y2) [33,34,35]. The 95% TI is an estimate of the central 95% range of population differences. If the TI is sufficiently narrow, then the two methods can be considered exchangeable. We defined “sufficiently narrow” as an exchangeability interval of (−1, 1), meaning XR-treatment and CT-treatment would be considered exchangeable if and only if the 95% TI was contained within (−1, 1).

The Bland–Altman method involved two estimates, along with their confidence interval (CI) variants. The estimates (lowercase) are estimates of population quantities (uppercase). Estimates are uncertain by their nature, and this uncertainty is incorporated into their CI variants. First, d = (m1 − m2) estimates the population quantity D = (M1 − M2), where (m1, m2) and (M1, M2) are the sample and population means of (y1, y2), respectively. The CI variant is the usual 95% CI, (d − 1.96 × se, d + 1.96 × se) indicating the plausible range for the population mean difference. The CI embodies the uncertainty in d via the standard error of d, se = s/sqrt(N), which gets smaller with larger N. Second, (d − 1.96 × s, d + 1.96 × s) estimates the population quantity (D − 1.96 × S, D + 1.96 × S), which we call the “prediction interval” (PI) (Bland and Altman’s “limits of agreement”). Here, S is the population standard deviation, which does not involve N. PI involves uncertainty in both d and s, and 95% CIs can be calculated for both lower and upper bounds, which incorporate this uncertainty. We are interested only in the lower confidence limit of PI’s lower bound and the upper confidence limit of PI’s upper bound. These are, respectively, the lower and upper bounds of the 95% tolerance interval, which indicates the plausible range for the central 95% of the population differences.

We conducted a standard Bland–Altman analysis, which does not involve predictors, as well as a regression generalization, which does. The predictors were MP along with three binary variables: (1) sex (0 for females; 1 for males); (2) age (0 for children < 10 years old; 1 for adolescents ≥ 10 years old); and (3) function score (FS) (0 for GMFCS = 1–2; 1 for GMFCS = 3–5), a grouping suggested by the 5 estimated profiles of d. To incorporate these predictors, Normal regression models were fit using a step function representing 10 MP levels: [0, 10], (10, 20], (20, 30], (30, 40], (40, 50], (50, 60], (60, 70], (70, 80], (80, 90], and (90, 100], along with indicator (dummy) variables for FS, sex, and age. This step function type of regression is highly local in the sense that an estimate for one step is not affected by estimates for other steps. In the regression context, the two Bland–Altman estimates (d, PI) and their CI variants (CI, TI) depend on predictor values, and such conditioning can be expected to provide better results (less bias and more precision) than the standard Bland–Altman analysis.

Models were estimated by maximum likelihood using the NLMIXED procedure of SAS 9.4 [36] and were assessed by AIC statistics as well as Wald and likelihood ratio tests [37]. Prediction intervals were calculated using quantile functions, and tolerance intervals were based on delta method approximations for the variance of these functions [38]. Because the standard deviation of d = (y1 − y2) was much lower for MP ≤ 20 than for MP > 20, different estimated standard deviations were used for the two regions.

## 3. Results

### 3.1. Study Cohort Characteristics

Our study cohort consisted of 139 children (male = 90; mean age = 9.3 ± 3.8 years) with 278 hips. The mean time interval between a child’s hip CT and the pelvic radiograph was 86 ± 52 days. The median GMFCS was 4. The clinical history entered by the ordering provider for the 139 hip CTs was distributed as follows: pre-surgical planning (N = 47), femoral version (N = 27), hip subluxation/dislocation (N = 24), hip dysplasia (N = 23), joint contracture (N = 4), limb length discrepancy (N = 1), hip alignment (N = 1), generalized dystonia (N = 1), acetabular impingement (N = 1), and none (N = 10).

The CT-treatment plans provided by the surgeon for the 278 hips in our study cohort were distributed as follows: conservative/preventive therapy (N = 96), femoral osteotomy (N = 63), femoral and pelvic osteotomies (N = 105), and salvage surgery (N = 14). In comparison, the XR-treatment plans determined based on the MP thresholds for the same 278 hips were distributed as follows: conservative/preventive therapy (N = 153), femoral osteotomy (N = 59), femoral and pelvic osteotomies (N = 36), and salvage surgery (N = 30).

### 3.2. Agreement of Treatment Plans

**Figure 2:**
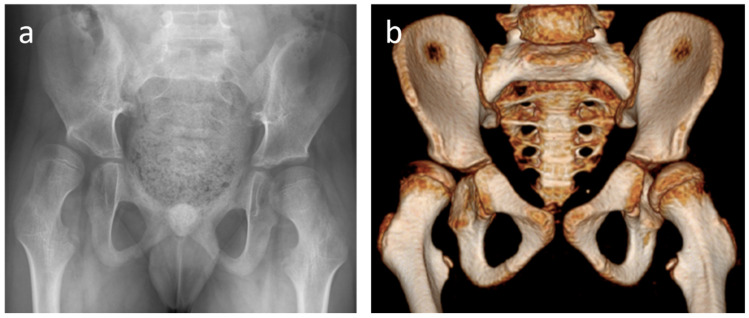
10-year-old boy with cerebral palsy. The right and left hip migration percentages (MPs), calculated from the screening pelvic radiograph (**a**), were 50% and 27%, respectively. Based on our institutional MP thresholds, the recommended treatment plans for the right and left hips would be femoral osteotomy and conservative/preventive therapy, respectively (XR-treatment). In contrast, the pediatric hip surgeon, after reviewing the hip CT (**b**), recommended femoral osteotomies for both hips (CT-treatment). In this patient, the XR- and CT-treatment plans agreed for the right hip but not for the left hip. In this case, the bilateral coxa valga deformities swayed the surgeon’s decision for bilateral femoral osteotomies, while the MPs did not take that information into account.

**Figure 3:**
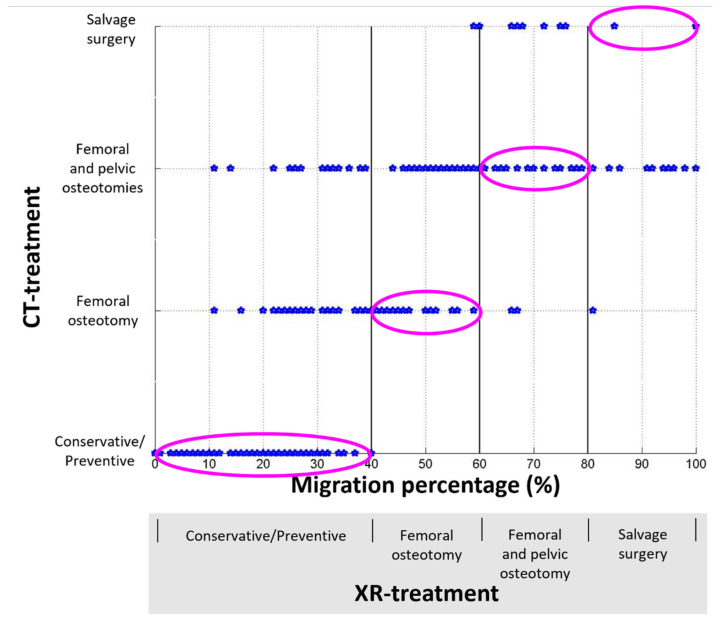
Scatter plot comparing the treatment plans made following review of the hip CTs by an orthopedic surgeon (CT-treatment) to those made based on migration percentages (MPs) derived from pelvic radiographs (XR-treatment). If XR-treatment and CT-treatment were in complete agreement with each other, the data points would all lie strictly within the pink ellipsoids, which is not the case here.

Figure 2 shows an example where the XR- and CT-treatments agreed for the right hip but not for the left hip. We used a scatter plot to visually illustrate the relationship between XR- and CT-treatments (Figure 3). We showed the 4 × 4 confusion matrix to highlight the similarities and differences between the XR- and CT-treatments (Table 1). The proportion of agreement, unweighted kappa, and weighted kappa between XR- and CT-treatment were 0.532 (148/278) (low), 0.339 (low), and 0.673 (moderate), respectively.

### 3.3. Exchangeability of Treatment Plans

**Figure 4:**
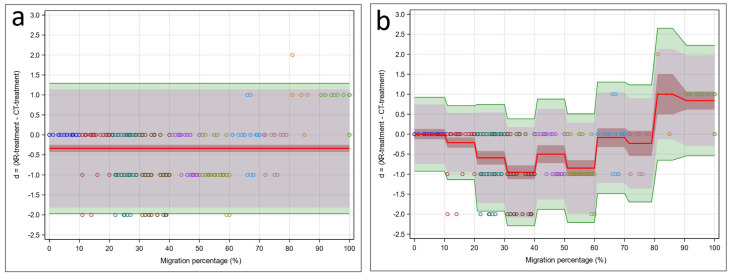
Bland–Altman models: (**a**) intercept model (no predictors) and (**b**) 10-step model involving the predictor MP. Observed differences between CT and X-ray (d = (y1 − y2)) are indicated by circles (colors indicate their MP range: [0, 10], (10, 20], …, (90, 100]). The difference d = (y1 − y2) estimates the population quantity D = (M1 − M2) (red line), with the 95% confidence interval (brown interval) indicating the plausible range for the population mean difference. The prediction interval (d − 1.96 × s, d + 1.96 × s) estimates the population quantity (D − 1.96 × S, D + 1.96 × S) (grey interval), with the confidence interval variant given by the tolerance interval (green interval), indicating the plausible range for the central 95% of the population differences.

The standard Bland–Altman method (no predictors) in Figure 4a was not suitable because of the strong effect of MP on the difference between XR-treatment (y1) and CT-treatment (y2) (*p* < 0.001). For this reason, we focus on the regression version of the Bland–Altman method (with predictor MP) in Figure 4b. This figure shows that the mean difference was zero in [0, 10], negative in (10, 80], and positive in (80, 100]. Thus, compared to CT-treatment, XR-treatment was similar in [0, 10], too low in (10, 80], and too high in (80, 100]. For this method comparison study, the location and width of the TIs were of particular interest. Based on our exchangeability interval of (−1, 1), XR-treatment and CT-treatment were exchangeable in [0, 10] but not in other intervals. XR-treatment was unacceptably low in (10, 80] and unacceptably high in (80, 100].

**Figure 5:**
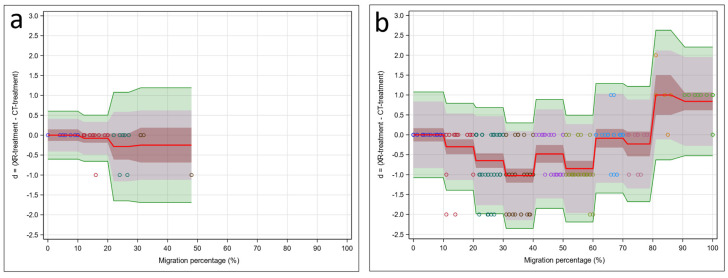
Separate Bland–Altman 10-step regression models for (**a**) FS = 0 (GMFCS 1 or 2) and (**b**) FS = 1 (GMFCS levels of 3, 4, or 5). Observed differences between CT and X-ray (d = (y1 − y2)) are indicated by circles (colors indicate their MP range: [0, 10], (10, 20], …, (90, 100]). The difference d = (y1 − y2) estimates the population quantity D = (M1 − M2) (red line), with the 95% confidence interval (brown interval) indicating the plausible range for the population mean difference. The prediction interval (d − 1.96 × s, d + 1.96 × s) estimates the population quantity (D − 1.96 × S, D + 1.96 × S) (grey interval), with the confidence interval variant given by the tolerance interval (green interval), indicating the plausible range for the central 95% of the population differences.

Regarding other predictors, for the single model applied to all subjects, neither sex (*p* = 0.962) nor age (*p* = 0.173) were significant, but function score (FS) was significant. The treatment difference d = (y1 − y2) was 0.23 units lower for FS = 1 than FS = 0 (*p* = 0.014). A likelihood ratio test indicated a preference for separate models for FS = 0 and FS = 1 (*p* = 0.001). Figure 5 shows the different profiles for separate models: FS = 0 and FS = 1 had very different MP ranges (FS = 0: range 0 to 50; FS = 1: range 0 to 100), as well as very different MP curves (the decline from 0 to 50 was much more pronounced for FS = 1 than FS = 0).

For separate models, MP had a weak effect on d = (y1 − y2) for FS = 0 (*p* = 0.011) and a strong effect for FS = 1 (*p* < 0.001). For both FS = 0 and FS = 1, the mean difference was zero in [0, 10], negative in (10, 80], and positive in (80, 100]. Thus, compared to CT-treatment, XR-treatment was similar in [0, 10], too low in (10, 80], and too high in (80, 100]. Based on an exchangeability interval of (−1, 1), XR-treatment and CT-treatment were exchangeable for MP ≤ 20 for FS = 0 but were not quite exchangeable for FS = 1. Neither sex nor age were significant for either separate model (*p*-values for male and age were 0.962 and 0.173 for FS = 0, and 0.622 and 0.647 for FS = 1, respectively).

## 4. Discussion

The traditional method for determining the treatment plans for hip displacements in children with CP has typically been based on MPs derived from AP pelvic radiographs [20]. However, we know very little regarding the adequacy of this method. Hence, we compared the traditional method to treatment plans made by an experienced pediatric CP hip surgeon following the review of the 3D hip CTs. Overall, we found that these two methods were exchangeable in cases where there was minimal subluxation and the MP ≤ 10%, but they were not exchangeable otherwise. This disagreement underscores the inherent limitation of relying on a ‘cookbook’ algorithm based on a single 2D metric for surgical planning, as it cannot account for the complex 3D acetabular and femoral pathomorphology that is fully appreciable on CT and critical for determining the precise surgical strategy. Under the assumption that CT-treatment is preferred to XR-treatment when these treatment plans differ, our results suggest using either XR-treatment or CT-treatment for MP ≤ 10% and using CT-treatment for MP > 10%.

This study was designed to isolate and compare the surgical planning information yielded by two common imaging modalities. By controlling for clinical variables, we demonstrate that the imaging modality itself is a major source of variance in the radiographic interpretation as it pertains to pre-surgical planning. In clinical practice, this imaging input is synthesized with the clinical examination while considering patient factors to tailor surgical decision-making for each child.

Pelvic radiograph is a fast and inexpensive diagnostic study for the assessment of hips. However, it is a 2D projectional image, which is associated with many inherent limitations (such as difficulties visualizing 3D bony details and accurately quantifying pathology). Further, the scalar metrics (such as MPs) derived from these pelvic radiographs for treatment planning are dependent on patient positioning and may not be accurate for all patients [30,39,40,41]. Importantly, the MPs derived from pelvic radiographs strictly measured the amount of acetabular coverage of the femoral head in the coronal plane without taking into consideration other factors (such as the overall morphology and spatial relationship of the acetabulum and the proximal femur). In short, MP is a suboptimal metric for the assessment of hip integrity when considering surgical intervention.

In contrast to pelvic radiography, hip CT (1) provides a comprehensive and detailed 3D description of the bony morphology for visualization and analysis; (2) is much less sensitive to patient positioning (due to multiplanar CT reformation capabilities); and (3) is less resource-intensive (since CT does not require a support team to hold the patient in the correct position). Though multiplanar CT has proven critical in achieving superior treatment outcomes in hip evaluations [42,43,44,45,46], it is not the standard-of-care for pre-surgical hip evaluation in children with CP. This may be partly due to the associated risks of increased radiation exposure with hip CTs, which can cause possible adverse effects to the growing skeleton. To overcome this barrier, we have adopted a novel CT imaging technique, based on X-ray spectral shaping via tin filtration, to substantially mitigate CT radiation exposure [47]. This technique has been shown to be successful in generating diagnostic quality hip CT in children with much reduced radiation exposure (comparable to that of a single pelvic radiograph with an effective dose of 0.28 ± 0.10 mSv per hip CT [48]).

A key question arising from our findings is related to clinical relevance: does the choice of imaging modality, and the consequent difference in surgical recommendation, affect patient outcomes? While our study design does not include outcome data, the direction of the observed discrepancies suggests important clinical implications based on established surgical principles. For hips in the 10–80% MP range, where XR-treatment was systematically less aggressive than CT-treatment, reliance on the MP algorithm alone risks under-treatment. This could mean performing an isolated femoral osteotomy when the acetabular dysplasia visible on CT necessitates a more aggressive combined femoral and pelvic osteotomies to achieve stability, potentially leading to residual subluxation, rapid arthrosis, and early revision surgery. Conversely, for hips with MP >80%, where XR-treatment was more aggressive (salvage), CT may identify a hip that is still reconstructible. Here, over-reliance on MP could lead to over-treatment, denying a patient a potentially successful joint-preserving reconstruction in favor of a salvage procedure with greater inherent morbidity and functional limitations. Therefore, the primary clinical relevance of our findings is that comprehensive 3D imaging can help tailor surgical intervention to the specific pathomorphology, potentially avoiding both under-correction and unnecessary salvage. This hypothesis—that CT-guided planning improves long-term surgical outcomes—warrants direct investigation in future prospective, longitudinal studies.

We must emphasize that both XR- and CT-treatment plans in this study represent imaging-based recommendations, which constitute only one element of the multifactorial decision-making process. The final treatment plan involves synthesis from imaging results, detailed clinical evaluation (including spasticity, contractures, pain, and function), and alignment with patient/family goals. Our finding that the imaging recommendations differ between modalities highlights the importance of the imaging component’s quality within the broader context of neuromuscular hip treatment. We believe this topic warrants further prospective exploration.

Despite the important conclusions obtained in our study, there are a few limitations which require acknowledgment. First, MP measurements were calculated by a single pediatric radiologist. Using average MPs calculated by a group of radiologists may yield more accurate results (although multiple studies in the literature have reported high inter-reader agreements for measuring MPs from radiographs [30,49,50]). Second, CT-treatments were derived based on the opinion of a single pediatric orthopedic surgeon. Having a consensus opinion from a group of hip surgeons to establish the most appropriate treatments would presumably increase precision (but also increase cost). Third, for the primary analysis, we treated individual hips as independent observational units. While hips from the same patient are anatomically and clinically correlated, our study design aimed to compare imaging modalities at the level of the hip joint, and dichotomizing each hip in a patient is methodologically sound approach. Fourth, our analysis employed a simplified four-tier treatment categorization. While this reflects a common conceptual framework for escalating intervention, it does not capture the full nuance of surgical planning and decision-making. This was a necessary simplification to enable a comparative statistical analysis, but it means our study evaluates agreement on the general stage or direction of treatment rather than on the precise surgical technique. Fifth, our study design pragmatically compared two clinical pathways: one based on a radiologist-provided metric from pelvic radiograph (XR-treatment) and another based on a surgeon’s assessment of the 3D hip CT (CT-treatment). Consequently, the imaging modality (radiograph versus CT) is confounded with the interpreter’s specialty (radiologist versus surgeon). This design was chosen to answer a clinical question about pathway-level differences. While it effectively simulates real-world decision-making, it cannot determine whether the observed discrepancies are driven primarily by the imaging technology, the interpreter’s expertise, or their interaction. A future study employing a crossed design could elegantly disentangle these factors.

## 5. Conclusions

We found that the decisions determined based on pelvic radiographs and those determined based on CTs were concordant only for those cases where MPs were small (MPs ≤ 10%). With increasing MPs and hip subluxations, the added information from the hip CTs can affect surgeon decision-making. Under the assumption that CT-treatment is preferred to XR-treatment when treatment plans differ, the suggestion is to use either XR-treatment or CT-treatment for MP ≤ 10% and to use CT-treatment for MP > 10%. Of course, it is also important to recognize that the ultimate treatment decision-making is multifactorial, including not only imaging interpretation but also clinical examination and patient/family factors.

## Figures and Tables

**Table 1:** Confusion matrix comparing the treatment plans made based on migration percentages (MPs) calculated from pelvic radiographs (XR-treatment) against treatment plans made following review of the hip CTs by an orthopedic surgeon (CT-treatment).

		CT-Treatment				
		Conservative/Preventive Therapy (Score = 0)	Femoral Osteotomy (Score = 1)	Femoral and Pelvic Osteotomies (Score = 2)	Salvage Surgery (Score = 3)	Total
XR-treatment	Conservative/preventive therapy (score = 0)	96	40	17	0	153
	Femoral osteotomy (score = 1)	0	20	37	2	59
	Femoral and pelvic osteotomies (score = 2)	0	2	27	7	36
	Salvage surgery (score = 3)	0	1	24	5	30
	Total	96	63	105	14	278

## Data Availability

The data that support the findings of this study are not openly available due to reasons of sensitivity and are available from the corresponding author upon reasonable request.
